# When the “Y” Becomes an “O”: A Narrative Review of Roux-en-O Complication After Roux-en-Y Gastric Bypass—Incidence, Diagnosis, Management, and Prevention

**DOI:** 10.1007/s11695-026-08660-y

**Published:** 2026-04-21

**Authors:** Hosam Hamed, Marwan Emara, Ahmed Farouk, Amr Sanad, Ibrahem Lotfy Abulazm, Mohamed Abdulrazek

**Affiliations:** Gastrointestinal Surgical Center, Almansoura, Egypt

## Abstract

Roux-en-O misconfiguration is a rare but serious technical error in Roux-en-Y gastric bypass (RYGB), resulting from the misidentification of small bowel limbs in which the biliopancreatic limb (BPL) is incorrectly anastomosed to the gastric pouch and the jejunojejunostomy (JJ) is misconfigured, leading to functional obstruction, bile reflux, and substantial morbidity. This narrative review synthesizes the available literature on its incidence, risk factors, clinical presentation, diagnostic challenges, differential diagnosis, treatment strategies, associated morbidity, and prevention methods. Drawing from case reports and small series, we highlight the diagnostic pitfalls and surgical corrections, including innovative approaches like jejunal interposition for complicated revisions. An illustrative case of a young woman with Roux-en-O following multiple bariatric conversions, complicated by a perigastric abscess, is integrated to demonstrate real-world application. Early recognition and meticulous intraoperative verification are essential to mitigate this preventable complication, preserving the efficacy of RYGB as a benchmark bariatric procedure.

**Supplementary Information:**

The online version contains supplementary material available at 10.1007/s11695-026-08660-y.

## Key Points

- Roux-en-O is a rare but serious misconstruction of Roux-en-Y gastric bypass that causes functional obstruction, bile reflux, and substantial morbidity.
- Roux-en-O diagnosis is challenging and often missed on imaging; definitive confirmation requires surgical exploration and careful tracing of bowel anatomy.
- Prevention of Roux-en-O depends on surgical expertise and intraoperative verification of limb identity by limb marking, short biliopancreatic limb, and anatomic tracing during surgery, especially in conversion procedures.

**Supplementary Information:**

The online version contains supplementary material available at 10.1007/s11695-026-08660-y.

## Introduction

The “Roux-en-Y” gastric bypass (RYGB) is the most effective and well-established surgical intervention for severe obesity because of its durable and effective outcomes. It is widely regarded as the benchmark procedure for evaluating other bariatric procedures [1].

The procedure combines restrictive and malabsorptive mechanisms [2]; restriction is achieved via a small gastric pouch (20–60 mL) separated from the remnant stomach [3], while malabsorption results from bypassing a segment of small bowel, including a Roux limb (100–200 cm) and a biliopancreatic limb (BPL) (50–150 cm) [4]. The Roux limb is anastomosed to the gastric pouch, and the BPL is anastomosed distally to the Roux limb, forming the Roux-en-Y configuration essential for the procedure’s success [5, 6].

Misidentification of small bowel loops can rarely lead to anastomosing the BPL to the gastric pouch, with the jejunojejunostomy (JJ) also misconstructed by anastomosing the Roux limb to the side of the BPL, resulting in a Roux-en-O configuration that creates a blind loop and functional obstruction [7]. This leads to antiperistaltic food transit, bile reflux, and obstruction, undermining the procedure’s efficacy. It has been described primarily in case reports and series [8–10], with no prior comprehensive literature reviews. This narrative review examines the literature on Roux-en-O, incorporating insights from case reports and series, and illustrates key concepts with a representative case to underscore clinical relevance.

## Methods

We conducted a narrative (non-systematic) review of the literature on Roux-en-O following RYGB. Sources included peer-reviewed journals indexed in PubMed/Medline and major bariatric and radiology journals (e.g., Obesity Surgery, SOARD, AJR, Eur J Radiol), textbook chapters, and society manuals from 2005 to 2025. Search terms combined “Roux-en-O”, “misconstruction”, “reverse peristalsis”, “biliopancreatic limb”, “jejunojejunostomy”, and “Roux-en-Y gastric bypass”. Reference lists of key articles were screened for additional citations. Given the rarity and heterogeneity of reports, formal meta-analysis was not feasible; evidence is summarized qualitatively and aligned with clinical experience.

### Incidence

Roux-en-O is exceedingly rare, documented mainly in isolated case reports and small series [8–10]. No large-scale epidemiological data exist, but one study on the learning curve for laparoscopic RYGB in a low-volume Asian center reported an incidence of 1 in 60 cases (1.67%) [11]. This suggests higher rates in inexperienced settings, though overall prevalence remains low due to the procedure’s standardization. Underreporting may contribute, as misconfigurations might be corrected intraoperatively without documentation or misattributed to other complications.

## Risk Factors

Several risk factors for Roux-en-O have been identified in the literature. Limited surgical experience is a significant risk factor, as inexperienced surgeons are more likely to misidentify the Roux and biliopancreatic limbs after division. This error is also more common in conversion procedures [7].

Additionally, a long BPL (> 75 cm) may be mistaken for the Roux limb, as it can readily reach the gastric pouch [8]. Such lengthening may be intended by the surgeon in high-BMI patients or a mistake due to misidentification of the ligament of Treitz, such as in intestinal malrotation, where the distal ileum is mistaken for it [7].

The surgical approach (open vs. laparoscopy) does not appear to significantly influence the incidence. There is a risk of intestinal loop misidentification during laparoscopy because of the limited view field, and the same may also occur with the open approach due to limited exposure secondary to the small midline incision and thick abdominal wall in such patients with obesity [8].

## Clinical Presentation

Patients with Roux-en-O typically present with symptoms mimicking small bowel obstruction, including nausea, abdominal pain, food intolerance, and bilious vomiting [12, 13]. Food must travel antiperistaltically through the BPL. The misconstructed JJ creates a pseudo-volvulus with the Roux limb twisting along its mesentery, and bile refluxes to the pouch, causing biliary gastritis. These mechanisms explain feeding difficulties and exaggerated weight loss [8], with disproportionate protein-calorie malnutrition compared to the typical postoperative RYGB course [14].

Although many patients report abdominal pain, emesis, or nausea post-bariatric surgery [15, 16], bilious vomiting should never be considered normal after RYGB and should raise suspicion for Roux-en-O misconstruction [8].

For example, in our illustrative case, a 20-year-old woman who underwent all procedures at private hospitals in Mansoura, Egypt. Her initial surgery was laparoscopic sleeve gastrectomy for severe obesity. Seven years later, it was converted to a laparoscopic one-anastomosis gastric bypass (OAGB) for recurrent weight gain. After one year, the patient had intractable biliary reflux with gastritis, which necessitated conversion to RYGB. Following RYGB, the patient developed persistent abdominal pain, recurrent bilious vomiting, and excessive weight loss. After nine months of unsuccessful medical management, she sought surgical consultation. Upper gastrointestinal endoscopy demonstrated excessive biliary secretions in the esophagus, gastric pouch, and alimentary limb extending to the JJ.

## Imaging and Diagnosis

Radiologic diagnosis is challenging, with plain abdominal X-rays, Computed tomography (CT), and fluoroscopy often appearing normal or nonspecific [8, 10]. CT may show features of proximal small bowel obstruction, such as Roux limb dilation [14, 17], and upper gastrointestinal fluoroscopy may reveal reverse Roux limb peristalsis [10, 17].

Technecium-99 m-labeled hydroxyiminodiacetic acid (HIDA) scintigraphy can be diagnostic [18]. Following Intravenous injection, the tracer is secreted by the liver into the duodenum; in a Roux-en-O, it travels via the BPL to the gastric pouch and esophagus, where its detection by a gamma camera confirms the diagnosis [8]. However, without suspicion, these findings may be overlooked. Definitive diagnosis typically requires surgical exploration with careful bowel tracing [7].

In the aforementioned case, CT volumetry revealed a hiatal hernia, shortened alimentary and biliopancreatic limbs (30 cm each), and a common channel length of 4.5 m. The preoperative plan was laparoscopic hiatal repair and JJ distalization. but Roux-en-O was confirmed laparoscopically after adhesiolysis and JJ division. Post-division, the presumed alimentary limb (attached to the gastric pouch) was found to be continuous to the ligament of Treitz, indicating it was the BPL mistakenly anastomosed to the gastric pouch by the prior surgeon and establishing a Roux‑en‑O misconstruction, explaining the patient’s symptoms.

## Differential Diagnosis

Roux-en-O must be differentiated from other causes of post-RYGB bilious vomiting, primarily common channel obstruction due to internal hernia, adhesions, or kinked JJ [7].

Intraoperatively, differentiating Roux-en-O from internal hernia is crucial, as tracing the loop attached to the gastric pouch may mislead the surgeon into thinking there is internal herniation, particularly when approaching the Treitz ligament. Expertise is crucial; in the illustrative case, initial suspicion of internal hernia shifted to Roux-en-O upon re-tracing post-JJ division.

### Treatment

As Roux-en-O is a technical misconfiguration, its treatment requires exploration (open or laparoscopic) and the creation of a correct RYGB configuration [9, 19]. Correction involves transecting the enteric segment between the gastric pouch and the JJ, then proceeding as in primary RYGB [9]. Another option is to divide both the gastrojejunostomy (GJ) and JJ, then create the correct “Roux-en-Y” pattern [13].

In complex cases with gastric pouch fibrosis, adaptations are needed. For instance, in the described case, challenges included the evacuated abscess related to the gastric pouch and a small, fibrotic pouch wall, which posed a high risk of leakage at a new GJ. Therefore, we interposed an isolated jejunal segment (originally attached to the gastric pouch) to elongate the short gastric pouch and provide a healthy wall for the new GJ. This novel modification may prove useful in similar scenarios with gastric pouch-related obstacles. A supplementary video demonstrating the laparoscopic revision for this illustrative case is attached (Supplementary Video).

Postoperatively, the patient experienced complete symptom resolution and resumed normal oral intake without vomiting. She was discharged on postoperative day two without complications.

## Morbidity After Surgical Correction

Correction of Roux-en-O carries increased risks of complications like anastomotic leak, aspiration pneumonia, deep venous thrombosis, intra-abdominal sepsis, and wound infection [7, 8]. Fortunately, our patient experienced an uneventful recovery. The illustrative patient’s smooth postoperative course underscores the feasibility of minimally invasive corrections.

## Prevention

Given its devastating impact, the prevention of this surgeon-related error is essential [18]. Suggested strategies include: (1) marking the Roux limb with a silk stitch or Penrose drain, (2) creating a short BPL (15–50 cm) as it will not easily reach the gastric pouch, or (3) tracing the bowel loop up to the Treitz ligament to confirm BPL identity [7, 8].

We propose an additional technique inspired by the concept of the “diverted OAGB” procedure. After gastric pouch creation, the bowel loop is anastomosed to it with the afferent limb (BPL) towards the left side and the efferent limb (alimentary) towards the right side. Now, the two loops are well identified. The afferent loop is divided just proximal to the GJ. Then, a JJ is created between the divided BPL and the alimentary limb distal to the GJ [20].

## Conclusion

Roux-en-O, a rare but preventable misconstruction that jeopardizes the safety and efficacy of RYGB. In patients with refractory bilious vomiting after RYGB, surgeons should maintain a high index of suspicion and confirm limb orientation intraoperatively. Prevention through meticulous anatomic verification is paramount; when revision is required in the setting of pouch fibrosis, a pouch-lengthening jejunal interposition offers a safe technical solution.

## Supplementary Information

Below is the link to the electronic supplementary material.

Supplementary Material 1

## Data Availability

No datasets were generated or analysed during the current study.
